# Organocatalytic Enantiospecific Total Synthesis of Butenolides

**DOI:** 10.3390/molecules26144320

**Published:** 2021-07-16

**Authors:** Rudrakshula Madhavachary, Rosy Mallik, Dhevalapally B. Ramachary

**Affiliations:** Catalysis Laboratory, School of Chemistry, University of Hyderabad, Central University (P.O.), Gachibowli, Hyderabad 500 046, India; m.c.rudrakshula@gmail.com (R.M.); mallikrosy@gmail.com (R.M.)

**Keywords:** butenolides, deoxygenation, reductive coupling, proline, total synthesis, organocatalysis

## Abstract

Biologically important, chiral natural products of butenolides, (−)-blastmycinolactol, (+)-blastmycinone, (−)-NFX-2, (+)-antimycinone, lipid metabolites, (+)-ancepsenolide, (+)-homoancepsenolide, mosquito larvicidal butenolide and their analogues were synthesized in very good yields in a sequential one-pot manner by using an organocatalytic reductive coupling and palladium-mediated reductive deoxygenation or organocatalytic reductive coupling and silica-mediated reductive deamination as the key steps.

## 1. Introduction

Developing a simple, sustainable, one-pot protocol by utilizing readily available starting materials for synthesizing a library of natural products, drugs and their analogues is a challenging task for the synthetic chemistry community [1,2,3]. The common drawbacks in the total synthesis of natural products and drugs are associated with multiple steps involving each step in column purification, leading to increases in solvent consumption, harmful wastes and decreases in yield. Furthermore, for the total synthesis of biologically important molecules to be industrially viable, use of toxic metal catalysts has to be avoided. Keeping these facts in mind, more attention has been devoted towards organocatalytic double domino, triple domino and quadruple domino sequential multi-step one-pot reactions. These sequential one-pot reactions can address the above cited problems by reducing the number of steps and thus minimizing the problems associated with them. That is why organocatalysis has been considered the green alternative route to the classical synthetic methods [4,5,6,7,8,9,10,11,12,13,14,15,16]. Even though there exist a considerable number of organo-catalytic asymmetric methods, only a few among them have made their way into the total synthesis of natural products and their analogs [17,18,19]. Among the known organocatalytic reactions, Michael [20,21,22], aldol [23,24], Diels–Alder [25] and Friedel–Crafts [26] reactions are the ones which have been repeatedly used in asymmetric total synthesis of natural products, drugs and drug intermediates.

Recently, our laboratory discovered the three-component organocatalytic reductive coupling (*OrgRC*) reaction for the selective *C*-alkylation of cyclic/acyclic CH-acids with different carbonyls in the presence of Hantzsch ester and this reaction has been utilized by many other synthetic chemistry groups in the total synthesis of natural products and drugs [27,28,29,30,31,32,33,34,35]. In our quest to develop organocatalytic methods and apply those methods in the total synthesis of natural products/drugs, we chose a medicinally important small library of natural products containing a butenolide (3-alkyl-5-methyl-2[5*H*] furanone) core as our synthetic target to synthesize through an organocatalytic sequential one-pot manner.

**Figure 1:**
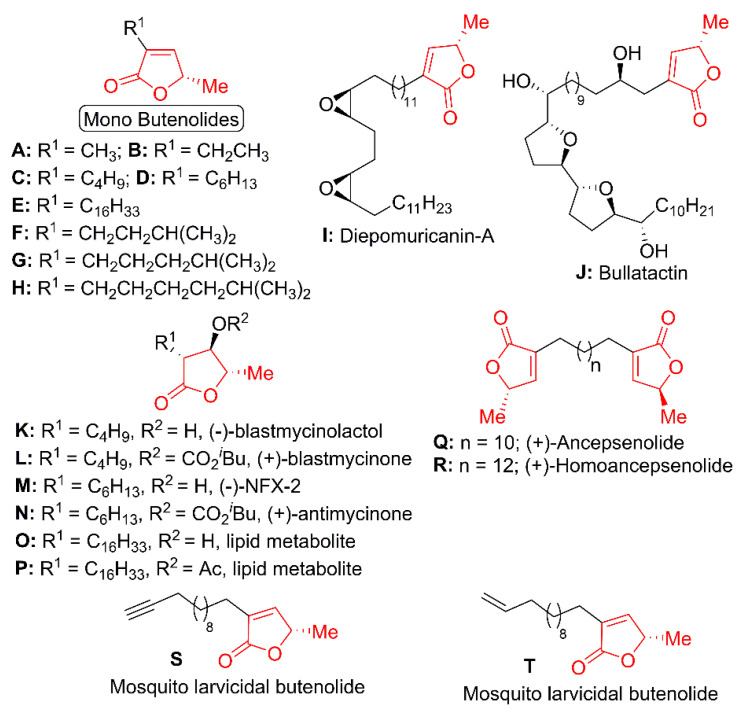
Butenolide natural products and their analogues.

Butenolides have attracted both isolation and synthetic chemists due to their broad range of bioactivities [36,37,38,39,40,41]. For example, butenolide **A** is a component in mushroom flavor [36], **B** shows fungicidal activity [37], **C**, **D**, **E**, **F**, **G** and **H** are *Streptomyces griseus* metabolites [38] and (+)-ancepsenolide **Q** and (+)-homoancepsenolide **R** have been known for their cytotoxicity and antimicrobial activity [39]. Structurally interesting diepomuricanin-A **I** and (+)-bullatacin **J** are effective cytotoxic acetogenins [40]. Functionalized butenolides such as (−)-blastmycinolactol **K**, (+)-blastmycinone **L**, (−)-NFX-2 **M** and (+)-antimycinone **N** are polyketide metabolites and show antifungal and antitumor properties, while **S** and **T** are mosquito larvicides [41] (Figure 1).

**Scheme 1:**
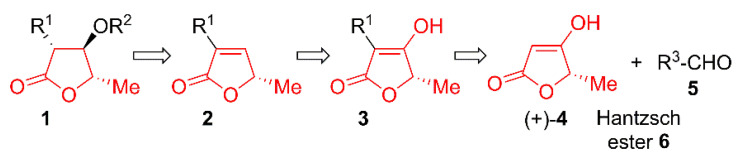
Retrosynthetic analysis for butenolides synthesis in a one-pot reaction.

Many of these butenolides **A**–**T** have been synthesized by using a combination of various metal-mediated dry reactions such as LDA-mediated diastereoselective alkylation/methylation, reductive deamination, asymmetric dihydroxylation, lactonisation, elimination, hydrotelluration, carbonyl reduction, enzymatic resolution, Te/Li exchange followed by lactonisation, aldol condensation followed by β-elimination, DCC-mediated coupling, reductive dehydroxylation or Ru-catalyzed olefin-alkyne Alder ene reaction as key steps [42,43,44,45,46,47,48,49,50,51,52,53,54,55,56,57,58,59,60]. Herein, we planned to use simple organocatalytic reactions with a combination of other simple reactions in a one-pot manner in ambient conditions to produce the butenolides in high yields. As outlined in Scheme 1, the alkylation at the 3-position of the known optically pure (*s*)-γ-methyl tetronic acid (+)-**4** could be done by using our organocatalytic reductive coupling (OrgRC) reaction, where an organic CH-acid (+)-**4** reacts with an aldehyde **5** to form in situ an olefin, which then spontaneously reacts with an organic hydrogen donor such as Hantzsch ester **6** in the presence of proline as a catalyst (Scheme 1). The 3-alkylated chiral tetronic acid **3** is a potent precursor for enantiospecific synthesis of the butenolide natural products **2** and **1** through simple transformations such as deoxygenation followed by epoxidation/ring-opening procedures [42,43,44,45,46,47,48,49,50,51,52,53,54,55,56,57,58,59,60]. This entire reaction sequence can be performed in a sequential one-pot manner to avoid wastage of solvents, time and to improve the overall process efficiency and the yield.

## 2. Results and Discussion

α,β-Unsaturated lactones are usually found in many natural products. In this context, butenolide natural products have a broad spectrum of biological activities. Many groups have already synthesized individual butenolide natural products using different methodologies in racemic and optically active forms [42,43,44,45,46,47,48,49,50,51,52,53,54,55,56,57,58,59,60]. Herein, we synthesized six of the natural products in the butenolide family by using the OrgRC reaction as the key step in the high-yield sequential one-pot process through C–C bond formation, and another six of them were made via formal total synthesis (see Figure 1 and Scheme 1).

Initially, we started the synthesis of monobutenolide natural products **C**, **D** and **E** from the chiral (*s*)-γ-methyl tetronic acid (+)-**4** and easily accessible alkyl aldehydes **5a**–**c**. The OrgRC reaction of (*s*)-γ-methyl tetronic acid (+)-**4** and aldehydes **5** in the presence of Hantzsch ester **6** under the proline-catalyst afforded the alkylated compounds **3** in excellent yields under ambient conditions. Then, the enol hydroxyl group of **3** was activated by converting to the corresponding *O*-triflate followed by palladium-catalyzed reductive deoxygenation for the preparation of monobutenolides **2** in excellent yields compared to previous reports [42,43,44,45,46,47,48,49,50,51,52,53,54,55,56,57,58,59,60].

**Scheme 2:**
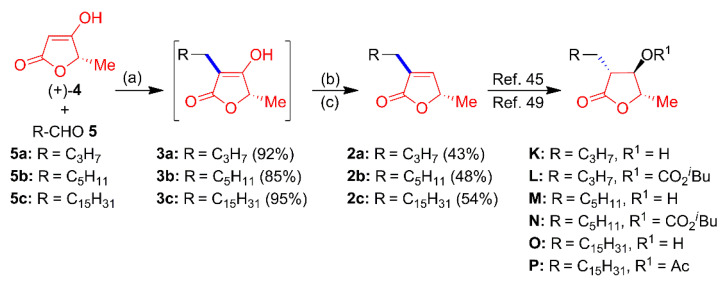
Synthesis of monobutenolides through sequential OrgRC/deoxygenation reactions in a one-pot method. Reagents and conditions: (a) **5** (1.0 equiv.), Hantzsch ester **6** (1.0 equiv.) relative to the (+)-**4**, proline (20 mol%), DCM (0.2 M), 25 °C, 2 h; (b) DIPEA (2.0 equiv.), Tf_2_O (1.5 equiv.), DCM (0.2 M), −78 °C, 1.0 h; (c) Pd(OAc)_2_ (10 mol%), DPPP (10 mol%), PMHS (2.0 equiv.), DMF (0.2 M), 60 °C, 8 h.

To reduce the number of purification steps, we tried to carry out OrgRC followed by *O*-triflate reaction in a one-pot manner. Hence, we preferred anhydrous CH_2_Cl_2_ as the solvent for OrgRC reaction which would also assist in the next step. The reaction of (*s*)-γ-methyl tetronic acid (+)-**4** with butyraldehyde **5a** and Hantzsch ester **6** under 20 mol% of proline in anhydrous CH_2_Cl_2_ at 25 °C for 2 h furnished the (*s*)-3-alkyl-4-hydroxy-5-methylfuran-2(5*H*)-one (+)-**3a** {[α]_D_^25^ = +1.2° (*c* = 0.51, CHCl_3_)} in very good yield/conversion. After the completion of the OrgRC reaction, two equiv. of *N*,*N*-diisopropylethylamine (DIPEA) was added to the reaction mixture, followed by 1.5 equiv. of Tf_2_O addition at −78 °C, and this was stirred for 1 h. After quenching the reaction with saturated NH_4_Cl solution and work-up, the crude product was used directly for the reductive deoxygenation reaction with Pd(OAc)_2_, 1,3-bis(diphenylphosphino)propane (DPPP) and polymethylhydrosiloxane (PMHS) in DMF at 60 °C for 8 h to furnish the desired monobutenolide (+)-**2a** and **C** {[α]_D_^25^ = +36.1° (*c* = 0.28, CHCl_3_), lit.: [45] [α]_D_^22^ = +79.2° (*c* = 1.18, CHCl_3_)} in 43% overall yield (Scheme 2). The same one-pot reaction sequence was executed with two more different aldehydes **5b** and **5c**, the monobutenolides (+)-**2b** and **D** {[α]_D_^25^ = +24.9° (*c* = 0.28, CHCl_3_)} and (+)-**2c** and **E** {[α]_D_^25^ = +24.8° (*c* = 0.5, CHCl_3_), lit.: [55] [α]_D_^25^ = +26.7° (*c* = 1.3, dioxane)} were obtained in 48% and 54% overall yields, respectively (Scheme 2). To our delight, the reagents and the by-products of OrgRC reaction did not affect the next reaction or the yield, but the final products (+)-**2a/C**, (+)-**2b/D** and (+)-**2c/E** required careful column-chromatography so as to separate them from the byproduct of Hantzsch ester pyridine. This present one-pot step-economy method seems to be better compared to the previous methods [42,43,44,45,46,47,48,49,50,51,52,53,54,55,56,57,58] with respect to the yields, reaction times and chemicals used (see Tables S1–S3, SI for correlation of steps and yields).

Chiral monobutenolides (+)-**2a/C**, (+)-**2b/D** and (+)-**2c/E** have their own biological activity and were also used as key intermediates in the expeditious asymmetric synthesis of antifungal and antitumor polyketide metabolites **K**-**P** by the Node and Comasseto groups [45,49]. (*s*)-3-Butyl-4-hydroxy-5-methylfuran-2(5*H*)-one (+)-**2a/C** is a potent precursor for synthesizing (−)-blastmycinolactol **K** and (+)-blastmycinone **L** following the reported procedures [45,49]. Following the same procedure, (−)-NFX-2 **M** and (+)-antimycinone **N** could be obtained from butenolide (+)-**2b/D** as the starting material [45,49]. In a similar manner, natural products **O** and **P** were synthesized by using (+)-**2c/E** as the starting material by the Font group [55]. Overall, our sequential one-pot method represents high-yield formal total synthesis of these chiral natural products **K** to **P** via OrgRC reaction as the key step.

**Scheme 3:**
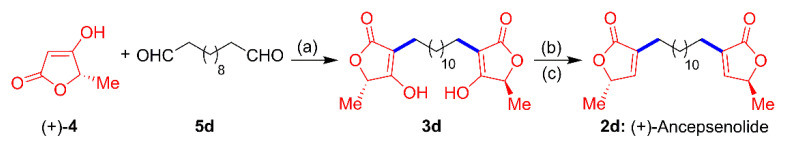
Total synthesis of (+)-ancepsenolide. Reagents and conditions: (a) (+)-**4** (2.0 equiv.), Hantzsch ester **6** (2.0 equiv.) relative to the **5d**, proline (20 mol%), DCM (0.2 M), 25 °C, 6 h; (b) DIPEA (2.0 equiv.), Tf_2_O (1.5 equiv.), DCM (0.2 M), -78 °C, 1 h; (c) Pd(OAc)_2_ (10 mol%), DPPP (10 mol%), PMHS (2.0 equiv.), DMF (0.2 M), 60 °C, 8 h, 55% overall yield in three steps.

Our next target was to synthesize (+)-ancepsenolide **Q** (isolated from gorgonian *pterogorgia anceps)*, an important *bis*-butenolide annonaceae acetogenin. The required 1,12-dodecanal **5d** was obtained from 1,12-dodecanol by PCC oxidation in quantitative yield. This was subjected to OrgRC reaction with chiral tetronic acid (+)-**4**, Hantzsch ester **6** and 20 mol% of proline. The reaction of **5d** with 2.0 equiv. of tetronic acid (+)-**4,** 2.0 equiv. of Hantzsch ester **6** in anhydrous DCM under proline (20 mol%) catalysis furnished the *bis*-alkylated product (+)-**3d** {[α]_D_^25^ = +18.3° (*c* = 0.14, MeOH)} in 99% conversion. Interestingly, the compound (+)-**3d** was highly polar and slightly insoluble in the reaction solvent DCM. After completion of the in situ OTf protection reaction, the solvent was evaporated and the crude solid material was dried and used for the next step as such. Following Pd-mediated reductive deoxygenation reaction sequence as above on the crude material, (+)-ancepsenolide (+)-**2d** and **Q** {[α]_D_^25^ = +30.5° (*c* = 0.12, CHCl_3_), lit.: [48] [α]_D_^25^ = +45.5° (*c* = 0.43, CHCl_3_)} was obtained in a satisfactory yield of 55% in overall three steps (Scheme 3). We are pleased to mention that this one-pot step-economy method is outstanding compared to the previous synthetic methods [42,43,44,45,46,47,48,49,50,51,52,53,54,55,56,57,58] with respect to the number of steps, nature of chemicals, yields and reaction times (see Table S4, SI for correlation).

**Scheme 4:**
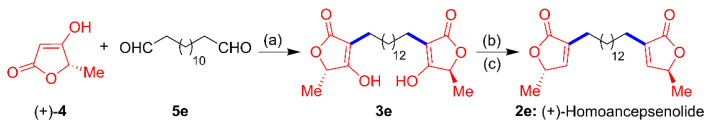
Total synthesis of (+)-homoancepsenolide. Reagents and conditions: (a) (+)-**4** (2.0 equiv.), Hantzsch ester **6** (2.0 equiv.) relative to the **5e**, proline (20 mol%), DCM (0.2 M), 25 °C, 6 h, 80% yield; (b) Pyrrolidine (1.5 equiv.), *p*-TSA (20 mol%), toluene, reflux, 15 h, 70% yield; (c) i. NaBH_3_CN (5.0 equiv.), MeOH, 2N HCl, 25 °C, 15 h, ii. SiO_2_ (2.0 equiv.), toluene, reflux, 7 h, 76% yield in 2 steps.

Furthermore, we accomplished the total synthesis of (+)-homoancepsenolide (+)-**2e** and **R** following the OrgRC/*O*-triflate and reductive deoxygenation reactions in a sequential one-pot manner, but unfortunately the desired *bis*-butenolide (+)-**2e** was isolated in poor yield. Hence, we followed Pashkovsky’s [47] approach for deoxygenation, by converting the bis-enol (+)-**3e** {[α]_D_^25^ = +1.2° (*c* = 0.51, CHCl_3_)} into the corresponding bis-enamine derivative followed by the reductive deamination through NaBH_3_CN-mediated reduction and SiO_2_-mediated amine-elimination to furnish the required (+)-homoancepsenolide (+)-**2e/R** {[α]_D_^25^ = +11.4° (*c* = 0.11, CHCl_3_)} in 42.5% with satisfactory overall yield (Scheme 4).

**Scheme 5:**
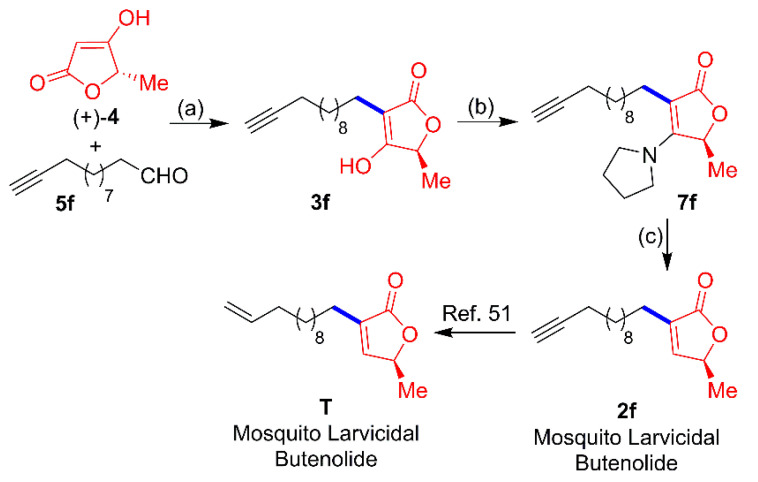
Total synthesis of mosquito larvicidal butenolides. Reagents and conditions: (a) (+)-**4** (1.0 equiv.), Hantzsch ester **6** (2.0 equiv.) relative to the **5f**, proline (20 mol%), DCM (0.2 M), 12 h, 25 °C, 90% yield; (b) Pyrrolidine (1.5 equiv.), *p*-TSA (20 mol%), toluene, reflux, 16 h, 85% yield; (c) i. NaBH_3_CN (5.0 equiv.), MeOH, 2N HCl, 15 h, 25 °C, ii. SiO_2_ (2.0 equiv.), toluene, reflux, 7 h, 38% yield in 2 steps.

The above results encouraged us to apply the OrgRC reaction for the synthesis of the butenolide **S**, an important natural product showing significant mosquito larvicidal properties (LC_50_ = 0.41 ppm), along with its partially reduced counterpart **T** (LC_50_ = 0.47 ppm) [40]. The required aldehyde **5f** was prepared from 1,10-decanediol in five simple steps. The chiral tetronic acid (+)-**4** and the aldehyde **5f** were subjected to OrgRC reaction conditions under proline catalysis in DCM to furnish the expected alkylated product (+)-**3f** {[α]_D_^25^ = +8.1° (*c* = 0.57, CHCl_3_)} in 90% yield. In our efforts to obtain the required deoxygenated product (+)-**2f** and **S**, we followed Pashkovsky’s approach. The desired OrgRC product (+)-**3f** was converted into the corresponding enaminone derivative **7f** followed by reducing the conjugated double bond and the elimination of the amine moiety furnished the required butenolide (+)-**2f** and **S** {[α]_D_^25^ = +30.6° (*c* = 0.11, CHCl_3_), lit.: [51] [α]_D_^25^ = +30.3° (*c* = 0.4, CHCl_3_)} in 30.6% overall yield (Scheme 5). This three-step method is shown to be better compared to the previous synthetic methods [51,59] with respect to the yields, reaction times and chemicals used (see Table S5, SI for correlation). Another important mosquito larvicide natural product **T** is the partially reduced counterpart of (+)-**2f/S** and can be obtained by simple functional group transformation as shown by Yao et al. [51].

We established the structure of the intermediates (+)-**3** and the natural products (+)-**2** by IR/NMR/mass analysis and the absolute configuration was confirmed by correlation with the published literature data including optical rotation (see SI).

## 3. Experimental Section

### 3.1. General Information

The ^1^H NMR and ^13^C NMR spectra were recorded at 400 or 500 MHz and 100 or 125 MHz, respectively. The chemical shifts are reported in ppm downfield to TMS (δ = 0) for ^1^H NMR and relative to the central CDCl_3_ resonance (δ = 77.0) for ^13^C NMR. In the ^13^C NMR spectra, the nature of the carbons (C, CH, CH_2_ or CH_3_) was determined by recording the DEPT-135 experiment and is given in parentheses. The coupling constants *J* are given in Hz. Column chromatography was performed using Acme’s silica gel (particle size 0.063–0.200 mm). High-resolution mass spectra were recorded on micromass ESI-TOF MS. IR spectra were recorded on JASCO FT/IR-5300 (JASCO Corporation, Tokyo, Japan) and Thermo Nicolet FT/IR-5700 (Thermo Electron Corporation, Waltham, Madison, USA). For thin-layer chromatography (TLC), silica gel plates Merck 60 F254 (Merck KGaA, Darmstadt, Germany) were used and compounds were visualized by irradiation with UV light and/or by treatment with a solution of *p*-anisaldehyde (23 mL), conc. H_2_SO_4_ (35 mL), acetic acid (10 mL) and ethanol (900 mL) followed by heating.

Experimental procedures and characterization data (^1^H NMR, ^13^C NMR and HRMS) can be found in Supplementary Materials.

### 3.2. Materials

All solvents and commercially available chemicals were used as received without further purification unless otherwise stated. Optically pure (*s*)-(+)-γ-methyltetronic acid **4** was prepared according to the literature procedure from (*s*)-(−)-ethyl lactate [61].

### 3.3. General Procedure for the Synthesis of OrgRC Products ***3***

A vial equipped with a magnetic stirbar containing proline (0.05 equiv.), (*s*)-(+)-γ-methyltetronic acid **4** (1.0 equiv) and Hantzsch ester **6** (1.0 equiv.) was charged with DCM (0.2 M), followed by addition of aldehyde **5a**–**f** (1.0 equiv.), and the resulting mixture was stirred at room temperature until the completion of the reaction as monitored by TLC. After completion of the OrgRC reaction, the organic layer was washed with brine, dried over Na_2_SO_4_ and concentrated. The crude product **3a**–**f** was used for the next step without purification.

### 3.4. General Procedure for the Synthesis of Products ***2*** through OrgRC and Palladium-Mediated Reductive Deoxygenation

**Step 1**: A vial equipped with a magnetic stirbar containing proline (0.05 equiv), (*s*)-(+)-γ-methyltetronic acid **4** (1 equiv) and Hantzsch ester **6** (1 equiv) was charged with DCM (0.2 M), followed by addition of aldehyde **5** (1 equiv), and the resulting mixture was stirred at room temperature until the completion of the reaction as monitored by TLC. After completion of the OrgRC reaction, DIPEA (2 equiv) was added to it. The reaction mixture was cooled to −78 °C and Tf_2_O (1.5 equiv, freshly distilled over P_2_O_5_) was added to it drop wise. After 30 min. (or completion of the reaction), the reaction mixture was quenched with saturated NH_4_Cl solution and partitioned between DCM (15 mL × 3) and water. The organic layer was washed with brine, dried over Na_2_SO_4_ and concentrated. The crude enol-triflate product was used for the next step without purification.

**Step 2**: General procedure for the palladium-catalyzed reduction of enol-triflate: The enol-triflate was dissolved in dry DMF (0.2 M). Pd(OAc)_2_ (0.01 mol%), 1,3-bis(diphenylphosphino)propane (DPPP) (0.01 mol %) and poly(methylhydrosiloxane) (PMHS) (2 equiv.) were added to the reaction mixture and heated at 60 °C. After completion of the reaction, the reaction mixture was diluted with diethyl ether and extracted with diethyl ether (10 mL × 3). The organic layer was washed with water, brine and dried over Na_2_SO_4_. After evaporation under reduced pressure, the crude compound **2** was purified by silica gel column chromatography.

### 3.5. General Procedure for the Synthesis of Products ***2*** through Silica-Mediated Reductive Deamination

**Step 1**: Compound **3** (1 equiv) was taken in a dry round bottom flask equipped with a Dean–Stark apparatus and reflux condenser in dry toluene (20 mL). Pyrrolidine (1.5 equiv) was added to it followed by catalytic *p*-TSA. The mixture was refluxed at 130 °C. After completion of the reaction, the mixture was concentrated and purified by column chromatography (neutral alumina) to get **7**.

**Step 2**: Procedure for the reduction of enaminone derivative: Compound **7** (1 equiv) was taken in MeOH and catalytic methyl orange was added to it. The mixture was acidified with a few drops of 2N HCl in MeOH so as to retain the deep red color of the indicator. NaBH_3_CN (2.5 equiv) was added in portions with simultaneous addition of acid to maintain the pH. After completion of the reaction, MeOH was removed under reduced pressure. The residue was diluted with water, neutralized with 1N NaOH and extracted with EtOAc (10 mL × 3). The combined organic layer was evaporated and the crude diastereomeric mixture of aminolactones was refluxed in toluene (6 mL) with silica gel for 7 h to get the product **2**.

## 4. Conclusions

In conclusion, we have developed a common methodology for the high-yield total synthesis of important butenolide natural products, from readily available simple substrates through the combination of organocatalytic reductive coupling and palladium-mediated reductive deoxygenation or organocatalytic reductive coupling and silica-mediated reductive deamination reaction sequences as the key steps. This high-yield sequential reductive protocol is an ideal method to synthesize the entire family of butenolide natural products.

## Supplementary Materials

The following are available online. Experimental procedures, and characterization data (^1^H NMR, ^13^C NMR, and HRMS) can be found in Supplementary Materials. Table S1: Yield comparative table of butenolide (+)-**2a**. Table S2: Yield comparative table of butenolide (+)-**2b**. Table S3: Yield comparative table of butenolide (+)-**2c**. Table S4: Yield comparative table of butenolide (+)-**2d**. Table S5: Yield comparative table of butenolide (+)-2**f**.
