# Supplementary material for: Organocatalytic Enantiospecific Total Synthesis of Butenolides
Source: Molecules. 2021 Jul 16;26(14):4320. doi: 10.3390/molecules26144320 (PMC8306825; doi:10.3390/molecules26144320)
Supplement: Supplementary file 1 [file molecules-26-04320-s001.zip › molecules-1272561-supplementary/Total Synthesis of Butenolides-SI-1-Revised.pdf]

Supporting Information-I

Organocatalytic Enantiospecific Total Synthesis of Butenolides

Rudrakshula Madhavachary,[a] Rosy Mallik,[a] and Dhevalapally B. Ramachary*

Catalysis Laboratory, School of Chemistry, University of Hyderabad, Central University (P.O.), Prof. CR Rao Road, Gachibowli, Hyderabad-500 046, Telangana, India

E-mail: ramsc@uohyd.ac.in and ramchary.db@gmail.com

[a] RMC and RM both are contributed equally to this work.

General Methods: The ^{1}H NMR and ^{13}C NMR spectra were recorded at 400 or 500 MHz and 100 or 125 MHz, respectively. The chemical shifts are reported in ppm downfield to TMS (δ = 0) for ^{1}H NMR and relative to the central $CDCl_3$ resonance (δ = 77.0) for ^{13}C NMR. In the ^{13}C NMR spectra, the nature of the carbons (C, CH, CH_2 or CH_3) was determined by recording the DEPT-135 experiment, and is given in parentheses. The coupling constants *J* are given in Hz. Column chromatography was performed using Acme's silica gel (particle size 0.063-0.200 mm). High-resolution mass spectra were recorded on micromass ESI-TOF MS. IR spectra were recorded on JASCO FT/IR-5300 and Thermo Nicolet FT/IR-5700. For thin-layer chromatography (TLC), silica gel plates Merck 60 F254 were used and compounds were visualized by irradiation with UV light and/or by treatment with a solution of *p*-anisaldehyde (23 mL), conc. H_2SO_4 (35 mL), acetic acid (10 mL), and ethanol (900 mL) followed by heating.

Materials: All solvents and commercially available chemicals were used as received without further purification unless otherwise stated. Optically pure (*S*)-(+)-γ-methyltetronic acid was prepared according to the literature procedure from (*S*)-(-)-ethyl lactate.[1]

Procedure-A: General procedure for the synthesis of TCRA products 3:

An vial equipped with a magnetic stirbar, containing proline (0.05 equiv.), (*S*)-(+)-γ-methyltetronic acid **4** (1.0 equiv) and Hantzsch ester **6** (1.0 equiv.) was charged with DCM (0.2 M), followed by addition of aldehyde **5a-f** (1.0 equiv.) and resulting mixture was stirred at room temperature till the completion of the reaction as monitored by TLC. After completion of TCRA reaction organic layer was washed with brine, dried over Na_2SO_4 and concentrated. The crude product **3a-f** was used for the next step without purification.

(*S*)-3-butyl-4-hydroxy-5-methylfuran-2(5*H*)-one (**3a**): Prepared by following the procedure **A** and purified by column chromatography using EtOAc/hexane and isolated as white powder. R_f = 0.3 (30% Ethyl acetate in Hexanes), Mp: 156 °C. **$[\alpha]_D^{25}$ = +1.2° (*c* = 0.514 g/100 mL, $CHCl_3$)**; IR (neat): ν_{max} 3377, 2953, 1731, 1649, 1463, 1205, 1068 and 827 cm^{-1}; ^1H NMR (400 MHz, $CDCl_3$) δ 4.83 (1H, q, *J* = 6.4 Hz), 2.20 (2H, t, *J* = 7.2 Hz), 1.50 (3H, d, *J* = 6.8 Hz), 1.45 (2H, pentet, *J* = 7.2 Hz), 1.31 (2H, pentet, *J* = 7.2 Hz), 0.90 (3H, t, *J* = 7.2 Hz); ^{13}C NMR ($CDCl_3$, DEPT-135) δ 177.7 (C), 177.4 (C), 100.9 (C), 75.3 (CH), 30.1 (CH_2), 22.4 (CH_2), 20.8 (CH_2), 17.8 (CH_3), 13.8 (CH_3); HRMS m/z 171.1021 [M + H], Calcd for $C_9H_{15}O_3$ 171.1021.

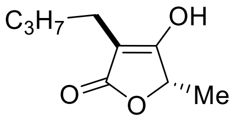

(+)-**3a**

(*S*)-3-hexyl-4-hydroxy-5-methylfuran-2(5*H*)-one (**3b**): Prepared by following the procedure **A** and purified by column chromatography using EtOAc/hexane and isolated as white powder. R_f = 0.3 (40% Ethyl acetate in Hexanes), Mp: 156 °C. **$[\alpha]_D^{25}$ = +10.6° (*c* = 0.50 g/100 mL, $CHCl_3$)**; IR (neat): ν_{max} 2932, 2863, 2639, 1732, 1458, 1379, 1217, 1128, 1071, 926 and 727 cm^{-1}; ^1H NMR (400 MHz, $CDCl_3$) δ 4.83 (1H, q, *J* = 6.8 Hz), 2.20 (2H, t, *J* = 7.2 Hz), 1.50 (3H, d, *J* = 6.8 Hz), 1.48-1.42 (2H, m), 1.32-1.23 (6H, m), 0.86 (3H, t, *J* = 7.2 Hz); ^{13}C NMR ($CDCl_3$) δ 178.0 (C), 177.8 (C), 100.7 (C), 75.4 (CH), 31.6 (CH_2), 29.0 (CH_2), 28.0 (CH_2), 22.6 (CH_2), 21.0 (CH_2), 17.8 (CH_3), 14.0 (CH_3); HRMS m/z 199.1334 [M + H]. Calcd for $C_{11}H_{19}O_3$ 199.1334.

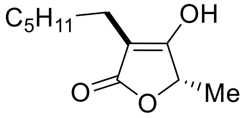

(+)-**3b**

(*S*)-3-hexadecyl-4-hydroxy-5-methylfuran-2(5*H*)-one (3**c**): Prepared by following the procedure **A** and purified by column chromatography using EtOAc/hexane and isolated as white powder. R_f = 0.3 (40% Ethyl acetate in Hexanes), Mp: 66 °C. **$[\alpha]_D^{25}$ = +3.9° (*c* = 0.428 g/100 mL, $CHCl_3$)**; IR (neat): ν_{max} 2919, 2849, 2721, 1711, 1634, 1470, 1269, 1084 and 837 cm^{-1}; ^1H NMR (500 MHz, $CDCl_3$) δ 4.83 (1H, q, *J* = 6.5 Hz), 2.20 (2H, t, *J* = 7.5 Hz), 1.51 (3H, d, *J* = 6.5 Hz), 1.49-1.43 (2H, m), 1.31-1.24 (26H, br m), 0.89 (3H, t, *J* = 6.5 Hz); ^{13}C NMR ($CDCl_3$) δ 177.4 (C), 177.1 (C), 101.1 (C), 75.2 (CH), 31.9 (CH_2), 29.7 (4 x CH_2), 29.68 (2 x CH_2), 29.66 (2 x CH_2), 29.5 (CH_2), 29.4 (CH_2), 29.3 (CH_2), 28.0 (CH_2), 22.7 (CH_2), 21.1 (CH_2), 17.8 (CH_3), 14.1 (CH_3); HRMS m/z 361.2715 [M + Na], Calcd for $C_{21}H_{38}O_3Na$ 361.2719.

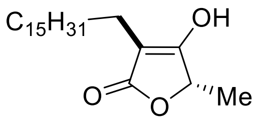

(+)-**3c**

(5*S*,5'*S*)-3,3'-(dodecane-1,12-diyl)bis(4-hydroxy-5-methylfuran-2(5*H*)-one) (3**d**): Prepared by following the procedure **A** and purified by column chromatography using EtOAc/hexane and isolated as white powder. R_f = 0.1 (Ethyl acetate), Mp: 138 °C. **$[\alpha]_D^{25}$ = +18.3° (*c* = 0.142 g/100 mL, MeOH)**; IR (neat): ν_{max} 2919, 2847, 2706, 1711, 1651, 1451, 1388, 1343, 1296, 1263, 1234, 1140, 1107, 1080, 1051, 918 and 789 cm^{-1}; ^1H NMR (400 MHz, CD_3OD) δ 4.77 (2H, q, *J* = 6.8 Hz), 2.15 (4H, t, *J* = 7.2 Hz), 1.50-1.44 (4H, m), 1.42 (6H, d, *J* = 6.8 Hz), 1.30 (16H, br s); ^{13}C NMR (CD_3OD) δ 178.4 (2 x C), 177.8 (2 x C), 101.0 (2 x C), 75.8 (2 x CH), 30.7 (4 x CH_2), 30.5 (2 x CH_2), 30.3 (2 x CH_2), 29.1 (2 x CH_2), 21.9 (2 x CH_2), 18.3 (2 x CH_3); HRMS m/z 417.2253 [M + Na], Calcd for $C_{22}H_{34}O_6Na$ 417.2253.

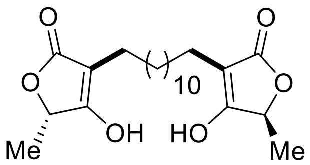

(+)-**3d**

(5*S*,5'*S*)-3,3'-(tetradecane-1,14-diyl)bis(4-hydroxy-5-methylfuran-2(5*H*)-one) (3**e**): Prepared by following the procedure **A** and purified by column chromatography using EtOAc/hexane and isolated as white powder. R_f = 0.1 (Ethyl acetate), Mp: 156 °C. **$[\alpha]_D^{25}$ = +1.2° (*c* = 0.514 g/100 mL, $CHCl_3$)**; IR (neat): ν_{max} 2918, 2847, 2702, 1711, 1647, 1452, 1386, 1342, 1284, 1255, 1140, 1107, 1080, 1051, 922. ^1H NMR (400 MHz,

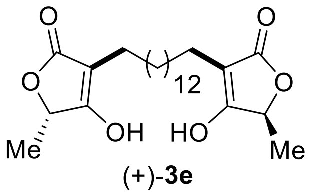

(+)-**3e**

$CDCl_3$ + CD_3OD) δ 4.51 (2H, q, J = 6.0 Hz), 2.00-1.96 (3H, m), 1.86-1.74 (1H, m), 1.31–1.14 (10H, m), 1.14–1.00 (20H, m); ^{13}C NMR ($CDCl_3$ + CD_3OD) δ 176.3 (2 x C), 176.2 (2 x C), 99.9 (2 x C), 74.1 (2 x CH), 29.2 (6 x CH_2), 29.0 (2 x CH_2), 28.9 (2 x CH_2), 27.6 (2 x CH_2), 20.6 (2 x CH_2), 17.3 (2 x CH_3). HRMS *m/z* Calcd for $C_{24}H_{38}O_6$: 422.2668; observed 423.2742 $[M + H]^+$, 445.2566 $[M + Na]^+$.

(*S*)-3-(dodec-11-yn-1-yl)-4-hydroxy-5-methylfuran-2(5*H*)-one (**3f**): Prepared by following

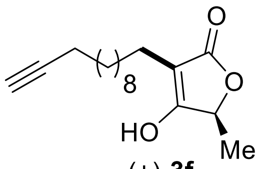

(+)-**3f**

the procedure **A** and purified by column chromatography using EtOAc/hexane and isolated as semisolid. R_f = 0.2 (30% Ethyl acetate in Hexanes); **$[\alpha]_D^{25}$ = +8.1° (*c* = 0.57 g/100 mL, $CHCl_3$)**; IR (neat): ν_{max} 3299, 2919, 2849, 1711, 1626, 1466, 1261, 1082, 843 and 638 cm^{-1}; ^{1}H NMR (400 MHz, $CDCl_3$) δ 4.84 (1H, q, J = 6.8 Hz), 2.21-2.15 (4H, m), 1.93 (1H, t, J = 2.4 Hz), 1.50 (3H, d, J = 6.8 Hz), 1.50-1.43 (4H, m), 1.39-1.36 (2H, m), 1.26 (10H, br s); ^{13}C NMR ($CDCl_3$) δ 178.0 (C), 177.8 (C), 100.7 (C), 84.7 (C), 75.4 (CH), 68.1 (CH), 29.6 (CH_2), 29.5 (CH_2), 29.4 (CH_2), 29.37 (CH_2), 29.1 (CH_2), 28.7 (CH_2), 28.4 (CH_2), 28.0 (CH_2), 21.0 (CH_2), 18.3 (CH_2), 17.8 (CH_3); HRMS m/z 301.1780 [M + Na], Calcd for $C_{17}H_{26}O_3Na$ 301.1780.

Procedure-B: General procedure for the synthesis of products 2:

Step-1: A vial equipped with a magnetic stirbar, containing proline (0.05 equiv), (*S*)-(+)-γ-methyltetronic acid **4** (1 equiv) and Hantzsch ester **6** (1 equiv) was charged with DCM (0.2 M), followed by addition of aldehyde **5** (1 equiv) and resulting mixture was stirred at room temperature till the completion of the reaction as monitored by TLC. After completion of TCRA reaction, DIPEA (2 equiv) was added to it. The reaction mixture was cooled to –78 ºC and Tf_2O (1.5 equiv, freshly distilled over P_2O_5) was added to it drop wise. After 30 min. (or completion of the reaction), the reaction mixture was quenched with saturated NH_4Cl solution and partitioned between DCM (15 mL x 3) and water. Organic layer was washed with brine, dried over Na_2SO_4 and concentrated. The crude enol-triflate product was used for the next step without purification.

Step-2: General procedure for the palladium catalyzed reduction of enol-triflate: The enol-triflate was dissolved in dry DMF (0.2 M). $Pd(OAc)_2$ (0.01 mol%), 1,3-bis(diphenylphosphino)propane (DPPP) (0.01 mol %) and poly(methylhydrosiloxane) (PMHS) (2 equiv.) were added to the reaction mixture and heated at 60 ºC. After completion of the reaction, the reaction mixture was diluted with diethyl ether and extracted with diethyl ether (10 mL x 3). The organic layer was washed with water, brine and dried over Na_2SO_4. After evaporation under reduced pressure, the crude compound **2** was purified by silica gel column chromatography.

Procedure-C: General procedure for the synthesis of products 2:

Step-1: Compound **3** (1 equiv) was taken in a dry round bottom flask equipped with a Dean-Stark apparatus and reflux condenser in dry toluene (20 mL). Pyrrolidine (1.5 equiv) was added to it followed by catalytic *p*-TSA. The mixture was refluxed at 130 ºC. After completion of the reaction, the mixture was concentrated and purified by column chromatography (neutral alumina) to get **7**.

Step-2: Procedure for the reduction of enaminone derivative: Compound **7** (1 equiv) was taken in MeOH and catalytic methyl orange was added to it. The mixture was acidified with a few drops of 2N HCl in MeOH so as to retain the deep red color of the indicator. $NaBH_3CN$ (2.5 equiv) was added in portions with simultaneous addition of acid to maintain the pH. After completion of the reaction, MeOH was removed under reduced pressure. The residue was diluted with water, neutralized with 1N NaOH and extracted with EtOAc (10 mL x 3). The combined organic layer was evaporated and crude diastereomeric mixture of aminolactones was refluxed in toluene (6 mL) with silica gel for 7 h to get the product **2**.

(*S*)-3-butyl-5-methylfuran-2(5*H*)-one (**2a**): Prepared by following the procedure **B** and

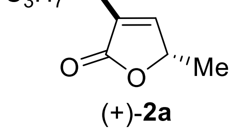

(+)-**2a**

purified by column chromatography using EtOAc/hexane and isolated as colorless oil. R_f = 0.3 (10% Ethyl acetate in Hexanes), **$[\alpha]_D^{25}$ = +36.1° (*c* = 0.28 g/100 mL, $CHCl_3$)**; IR (neat): ν_{max} 3362, 2934, 2878, 1755, 1605, 1454, 1321, 1200, 1113, 1028 and 875 cm^{-1}; ^1H NMR (500 MHz, $CDCl_3$) δ 7.00 (1H, q, *J* =

2.0 Hz), 5.00 (1H, qq, *J* = 2.0, 8.5 Hz), 2.28 (2H, tt, *J* = 2.0, 9.5 Hz), 1.58-1.50 (2H, m), 1.41 (3H, d, *J* = 8.5 Hz), 1.38-1.32 (2H, m), 0.93 (3H, t, *J* = 9.5 Hz); ^{13}C NMR ($CDCl_3$) δ 173.9 (C), 148.9 (CH), 134.2 (C), 77.4 (CH), 29.5 (CH_2), 24.8 (CH_2), 22.2 (CH_2), 19.1 (CH_3), 13.7 (CH_3); HRMS m/z 155.1072 [M + H], Calcd for $C_9H_{15}O_2$ 155.1072.

(*S*)-3-hexyl-5-methylfuran-2(5*H*)-one (**2b**): Prepared by following the procedure **B** and purified by column chromatography using EtOAc/hexane and isolated as sticky solid. R_f = 0.3 (10% Ethyl acetate in Hexanes), **$[\alpha]_D^{25}$ = +24.9° (*c* = 0.28 g/100 mL, $CHCl_3$)**; IR (neat): ν_{max} 2926, 2855, 1759, 1655, 1463, 1381, 1320, 1118, 1074, 1025 and 866 cm^{-1}; ^{1}H NMR (500 MHz, $CDCl_3$) δ 7.00 (1H, q, *J* = 2.0 Hz), 5.00 (1H, qq, *J* = 8.5, 2.0 Hz), 2.26 (2H, tt, *J* = 9.0, 2.5 Hz), 1.58-1.51 (2H, m), 1.41 (3H, d, *J* = 8.5 Hz), 1.35-1.25 (6H, m), 0.88 (3H, t, *J* = 8.5 Hz); ^{13}C NMR ($CDCl_3$) δ 173.9 (C), 148.9 (CH), 134.2 (C), 77.4 (CH), 31.4 (CH_2), 28.8 (CH_2), 27.3 (CH_2), 25.1 (CH_2), 22.5 (CH_2), 19.1 (CH_3), 14.0 (CH_3); HRMS m/z 183.1385 [M + H], Calcd for $C_{11}H_{19}O_2$ 183.1385.

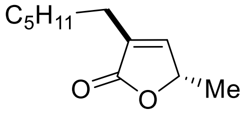

(+)-**2b**

(*S*)-3-hexadecyl-5-methylfuran-2(5*H*)-one (**2c**): Prepared by following the procedure **B** and purified by column chromatography using EtOAc/hexane and isolated as white powder. R_f = 0.3 (5% Ethyl acetate in Hexanes), Mp: 59 °C. **$[\alpha]_D^{25}$ = +24.8° (*c* = 0.5 g/100 mL, $CHCl_3$)**; IR (neat): ν_{max} 3079, 2917, 2849, 1746, 1651, 1472, 1373, 1331, 1201, 1120, 1082, 1030, 883 and 638 cm^{-1}; ^{1}H NMR (500 MHz, $CDCl_3$) δ 7.00 (1H, q, *J* = 1.5 Hz), 5.00 (1H, qq, *J* = 6.5, 1.5 Hz), 2.27 (2H, tt, *J* = 7.5, 1.5 Hz), 1.55 (2H, quintet, *J* = 7.5 Hz), 1.41 (3H, d, *J* = 6.5 Hz), 1.34-1.24 (26H, m), 0.88 (3H, t, *J* = 6.5 Hz); ^{13}C NMR ($CDCl_3$) δ 173.8 (C), 148.8 (CH), 134.3 (C), 77.3 (CH), 31.9 (CH_2), 29.65 (4 x CH_2), 29.61 (2 x CH_2), 29.6 (CH_2), 29.5 (CH_2), 29.31 (CH_2), 29.3 (CH_2), 29.1 (CH_2), 27.4 (CH_2), 25.1 (CH_2), 22.6 (CH_2), 19.2 (CH_3), 14.0 (CH_3); HRMS m/z 323.2950 [M + H], Calcd for $C_{21}H_{39}O_2$ 323.2950.

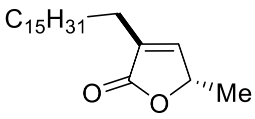

(+)-**2c**

(5*S*,5'*S*)-3,3'-(dodecane-1,12-diyl)bis(5-methylfuran-2(5*H*)-one) (**2d**): Prepared by following the procedure **B** and purified by column chromatography using EtOAc/hexane and isolated as white powder. R_f = 0.3 (10% Ethyl acetate in Hexanes), Mp: 97 °C. **$[\alpha]_D^{25}$ = +30.5° (*c* = 0.12 g/100 mL, $CHCl_3$)**; IR (neat): ν_{max} 2913, 2846, 1744, 1650, 1469, 1330, 1205, 1029 and 879 cm^{-1}; ^1H NMR (400 MHz, $CDCl_3$) δ 7.00 (2H, q, *J* = 1.6 Hz), 5.00 (2H, qq, *J* = 6.8, 1.6 Hz), 2.27 (4H, tt, *J* = 7.2, 1.6 Hz), 1.55 (4H, quintet, *J* = 7.6 Hz), 1.42 (6H, d, *J* = 6.8 Hz), 1.35-1.26 (16H, m); ^{13}C NMR ($CDCl_3$) δ 173.9 (2 x C), 148.9 (2 x CH), 134.3 (2 x C), 77.4 (2 x CH), 29.5 (2 x CH_2), 29.4 (2 x CH_2), 29.3 (2 x CH_2), 29.1 (2 x CH_2), 27.4 (2 x CH_2), 25.1 (2 x CH_2), 19.2 (2 x CH_3); HRMS m/z 363.2535 [M + H], Calcd for $C_{22}H_{35}O_4$ 363.2535.

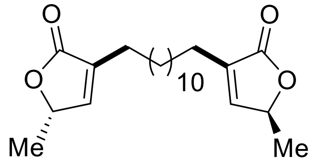

(+)-**2d:** (+)-Ancepsenolide

(5*S*,5'*S*)-3,3'-(tetradecane-1,14-diyl)bis(5-methylfuran-2(5*H*)-one) (**2e**): Prepared by following the procedure **C** and purified by column chromatography using EtOAc/hexane and isolated as white powder. R_f = 0.3 (30% Ethyl acetate in Hexanes), Mp: 100 °C. **$[\alpha]_D^{25}$ = +11.4° (*c* = 0.11 g/100 mL, $CHCl_3$)**; IR (neat): ν_{max} 2922, 2851, 1746, 1653, 1472, 1321, 1086, 1028 and 889 cm^{-1}; ^1H NMR (400 MHz, $CDCl_3$) δ 6.99 (2H, q, *J* = 1.6 Hz), 5.00 (2H, qq, *J* = 6.8, 1.6 Hz), 2.27 (4H, tt, *J* = 9.2, 1.6 Hz), 1.55 (4H, quintet, *J* = 7.6 Hz), 1.41 (6H, d, *J* = 6.8 Hz), 1.36-1.21 (20H, m); ^{13}C NMR ($CDCl_3$) δ 173.9 (2 x C), 148.9 (2 x CH), 134.3 (2 x C), 77.4 (2 x CH), 29.7 (2 x CH_2), 29.6 (2 x CH_2), 29.5 (2 x CH_2), 29.3 (2 x CH_2), 29.2 (2 x CH_2), 27.4 (2 x CH_2), 25.2 (2 x CH_2), 19.2 (2 x CH_3); HRMS m/z 391.2848 [M + H], Calcd for $C_{24}H_{39}O_4$ 391.2848.

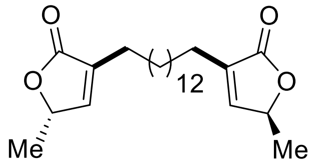

(+)-**2e:** (+)-Homoancepsenolide

(*S*)-3-(dodec-11-yn-1-yl)-5-methylfuran-2(5*H*)-one (**2f**): Prepared by following the procedure **C** and purified by column chromatography using EtOAc/hexane and isolated as oily liquid. Rf. = 0.2 (15% Ethyl acetate in Hexanes), **$[\alpha]_D^{25}$ = +30.6° (*c* = 0.11 g/100 mL, $CHCl_3$)**; IR (neat): ν_{max} 3308, 2928, 2855, 2116, 1755, 1655, 1462, 1373, 1319, 1200, 1076, 1026, 866 and 623 cm^{-1}; ^1H NMR (500 MHz, $CDCl_3$) δ 7.00 (1H, q, *J* = 1.5 Hz),

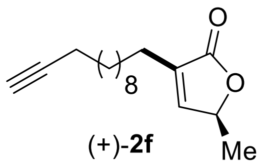

(+)-**2f**

5.00 (1H, qq, J = 6.5, 1.5 Hz), 2.27 (2H, tt, J = 7.5, 1.5 Hz), 2.19 (2H, td, J = 7.0, 2.5 Hz), 1.94 (1H, t, J = 2.5 Hz), 1.56-1.50 (4H, m), 1.42 (3H, d, J = 7.0 Hz), 1.41-1.36 (2H, m), 1.35-1.29 (10H, m); ^{13}C NMR ($CDCl_3$) δ 173.9 (C), 148.9 (CH), 134.3 (C), 84.7 (C), 77.4 (CH), 68.0 (CH), 29.4 (2 x CH_2), 29.2 (CH_2), 29.1 (CH_2), 29.0 (CH_2), 28.7 (CH_2), 28.4 (CH_2), 27.4 (CH_2), 25.1 (CH_2), 19.2 (CH_3), 18.3 (CH_2); HRMS 285.1833 [M + Na], m/z Calcd for $C_{17}H_{26}O_2Na$ 285.1831.

References:

1) S. Brandange, L. Flodman, and A. Norberg, *J. Org. Chem.* 1984, **49**, 927-928.
