# Supplementary material for: Organocatalytic Enantiospecific Total Synthesis of Butenolides
Source: Molecules. 2021 Jul 16;26(14):4320. doi: 10.3390/molecules26144320 (PMC8306825; doi:10.3390/molecules26144320)
Supplement: Supplementary file 1 [file molecules-26-04320-s001.zip › molecules-1272561-supplementary/Total Synthesis of Butenolides-SI-2-Revised.pdf]

Supporting Information-II
NMR Spectral Data

Organocatalytic Enantiospecific Total Synthesis of Butenolides

Rudrakshula Madhavachary,[a] Rosy Mallik,[a] and Dhevalapally B. Ramachary*

Catalysis Laboratory, School of Chemistry, University of Hyderabad, Central University (P.O.), Prof. CR Rao Road, Gachibowli, Hyderabad-500 046, Telangana, India

E-mail: ramsc@uohyd.ac.in and ramchary.db@gmail.com

[a] RMC and RM both are contributed equally to this work.

General Methods: The ^{1}H NMR and ^{13}C NMR spectra were recorded at 400 or 500 MHz and 100 or 125 MHz, respectively. The chemical shifts are reported in ppm downfield to TMS (δ = 0) for ^{1}H NMR and relative to the central $CDCl_3$ resonance (δ = 77.0) for ^{13}C NMR. In the ^{13}C NMR spectra, the nature of the carbons (C, CH, CH_2 or CH_3) was determined by recording the DEPT-135 experiment, and is given in parentheses. The coupling constants *J* are given in Hz. Column chromatography was performed using Acme's silica gel (particle size 0.063-0.200 mm). High-resolution mass spectra were recorded on micromass ESI-TOF MS. IR spectra were recorded on JASCO FT/IR-5300 and Thermo Nicolet FT/IR-5700. The X-ray diffraction measurements were carried out at 298 K on an automated Enraf-Nonious MACH 3 diffractometer using graphite monochromated, Mo-Kα (λ = 0.71073 Å) radiation with CAD4 software or the X-ray intensity data were measured at 298 K on a Bruker SMART APEX CCD area detector system equipped with a graphite monochromator and a Mo-Kα fine-focus sealed tube (λ = 0.71073 Å). For thin-layer chromatography (TLC), silica gel plates Merck 60 F254 were used and compounds were visualized by irradiation with UV light and/or by treatment with a solution of *p*-anisaldehyde (23 mL), conc. H_2SO_4 (35 mL), acetic acid (10 mL), and ethanol (900 mL) followed by heating.

Materials: All solvents and commercially available chemicals were used as received without further purification unless otherwise stated. Optically pure (*S*)-(+)-γ-methyltetronic acid was prepared according to the literature procedure from (*S*)-(-)-ethyl lactate.[1]

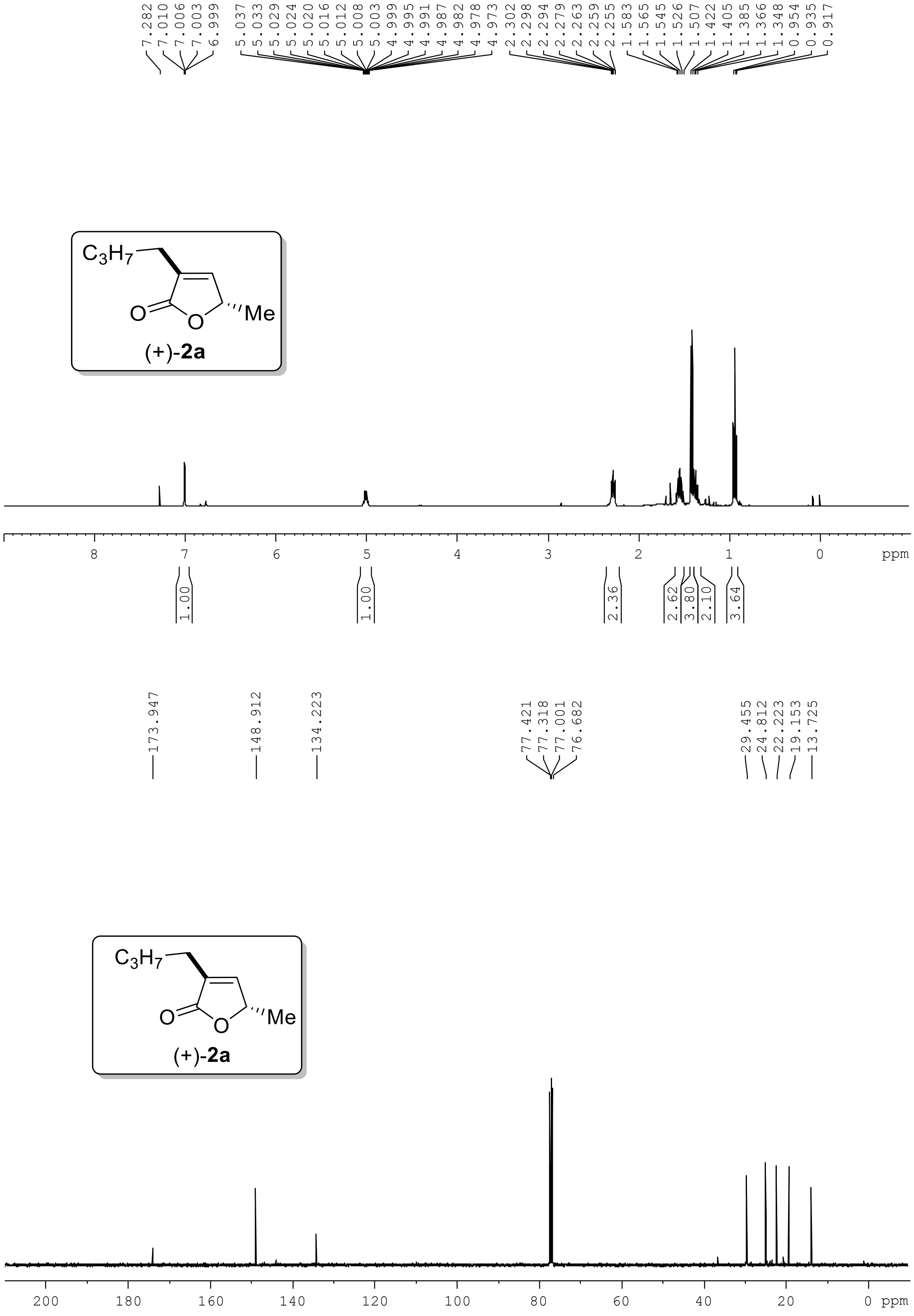

Figure-S1. ^{1}H and ^{13}C NMR spectra of the product (+)-**2a**.

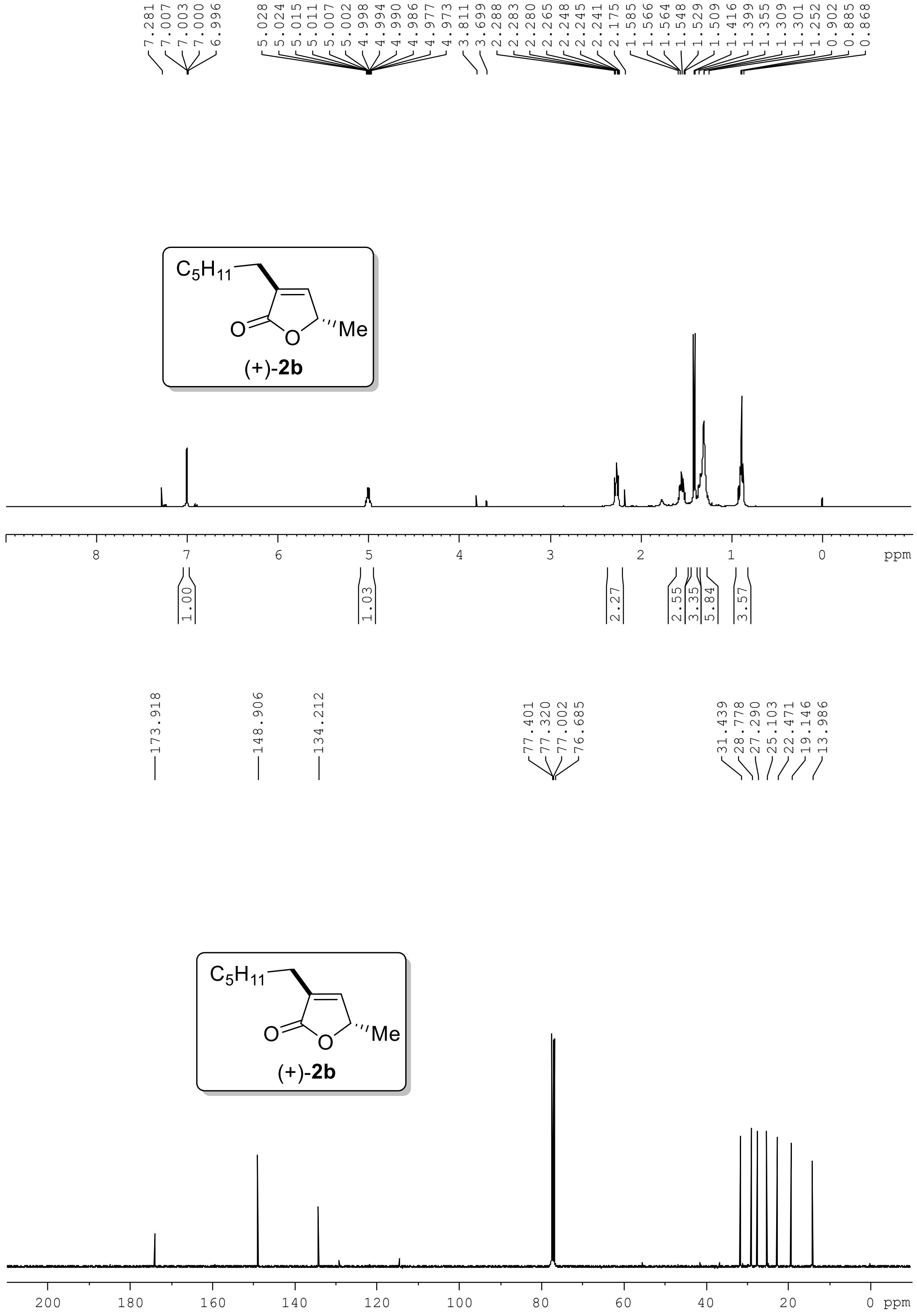

Figure-S2. ^{1}H and ^{13}C NMR spectra of the product (+)-**2b**.

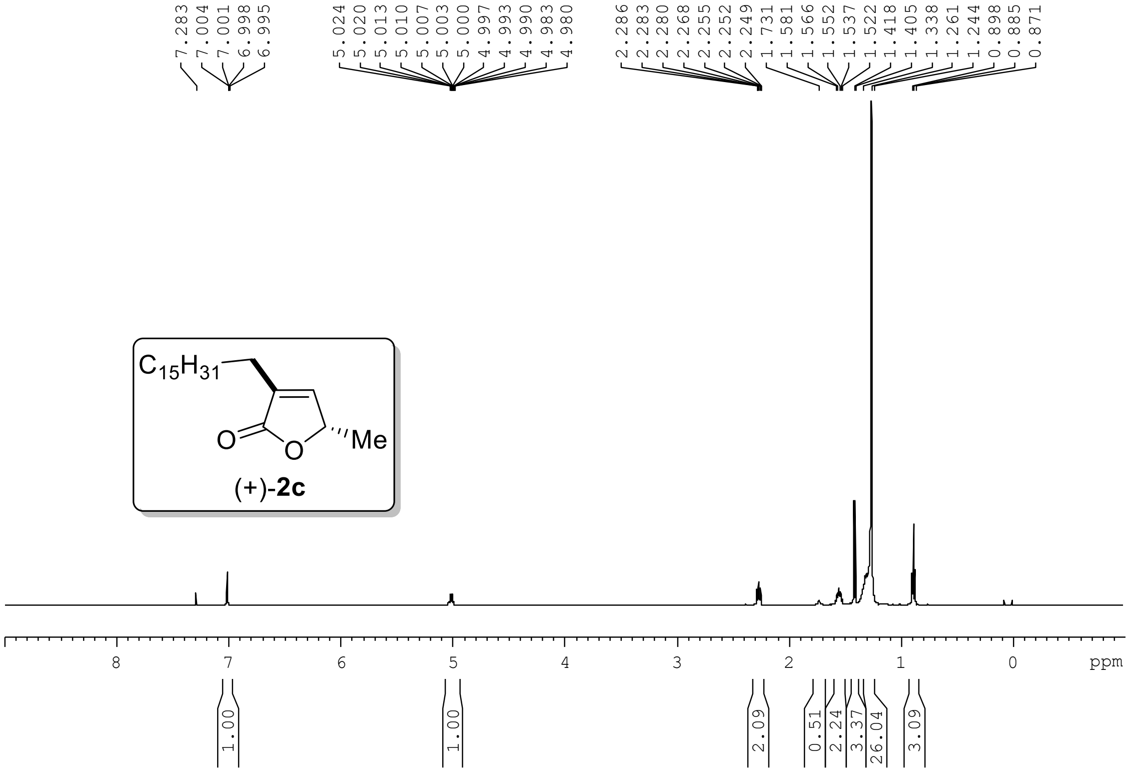

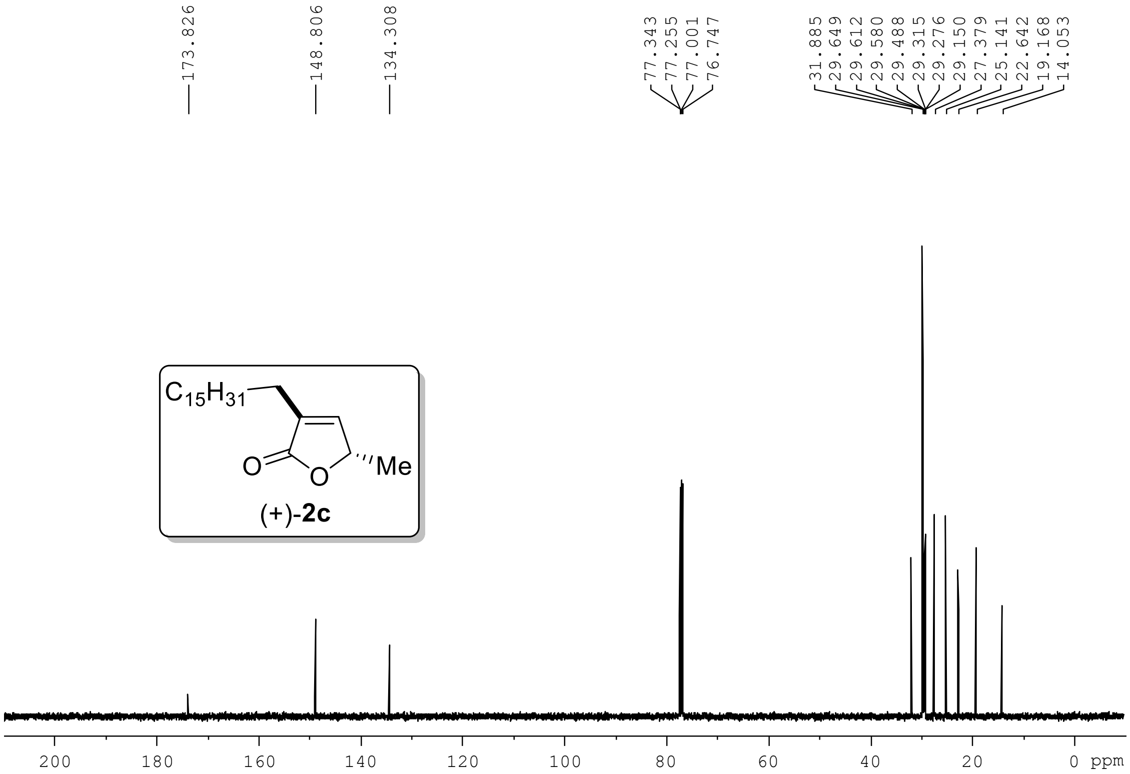

Figure-S3. ^{1}H and ^{13}C NMR spectra of the product (+)-**2c**.

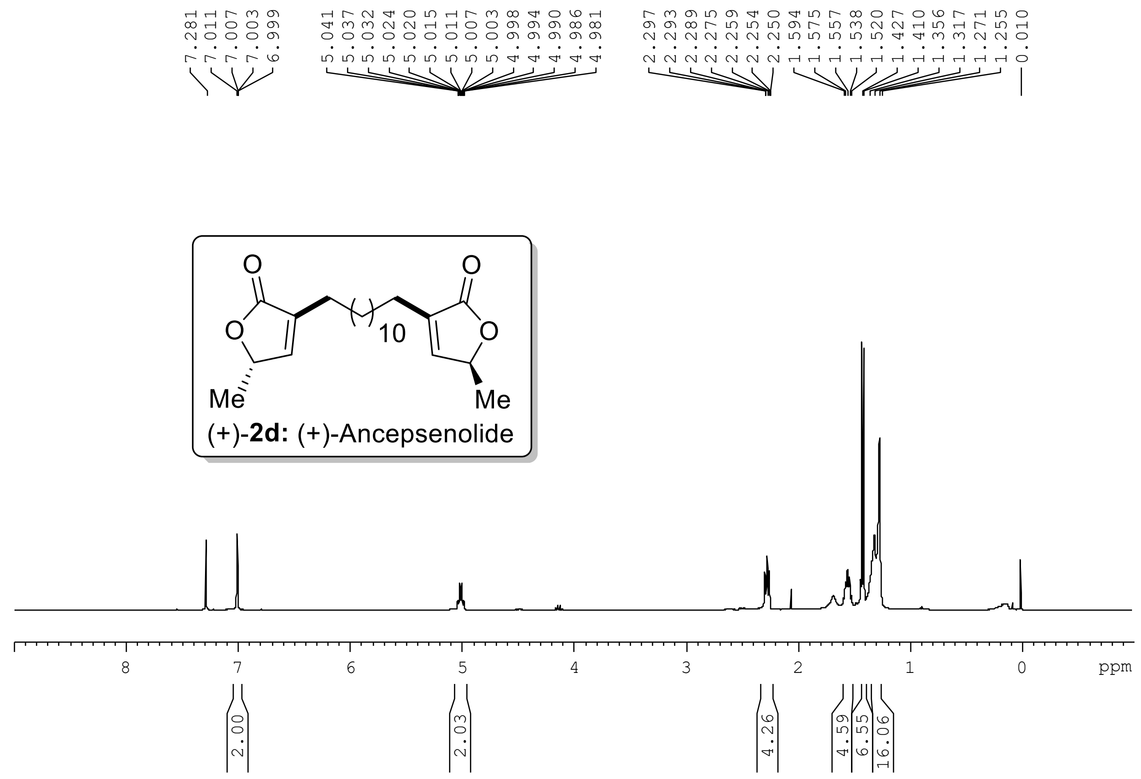

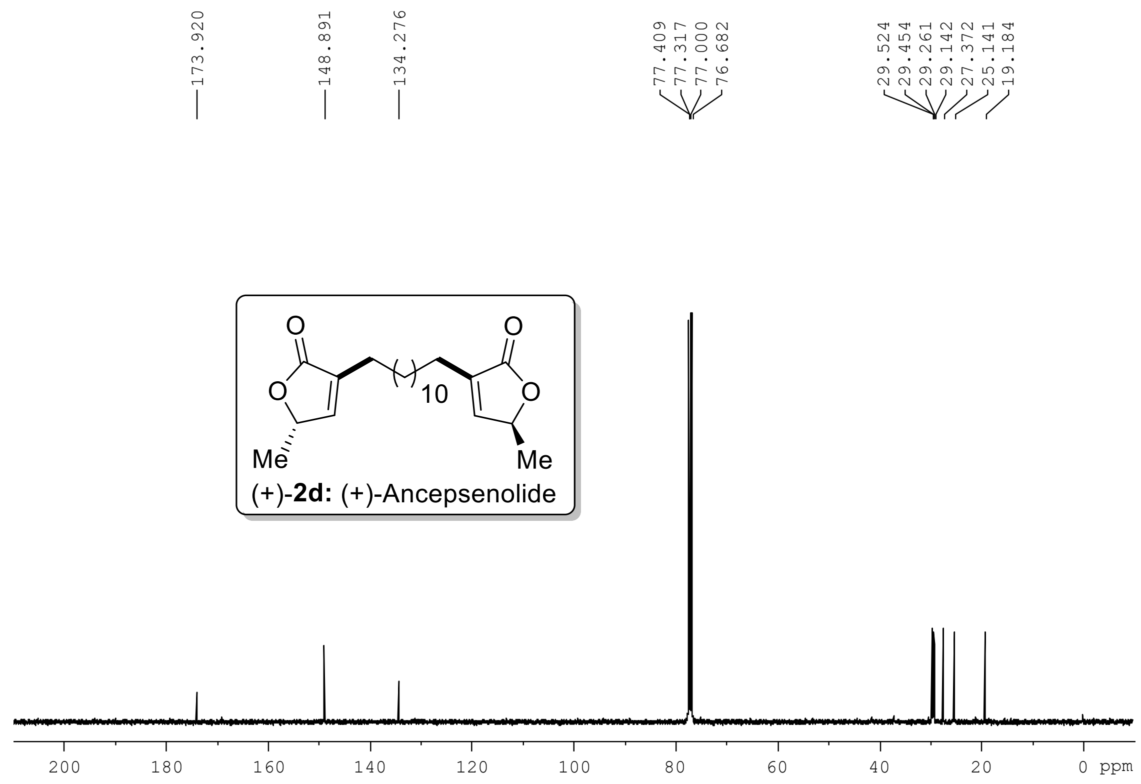

Figure-S4. ^{1}H and ^{13}C NMR spectra of the product (+)-**2d** **[**(+)-Ancepsenolide].

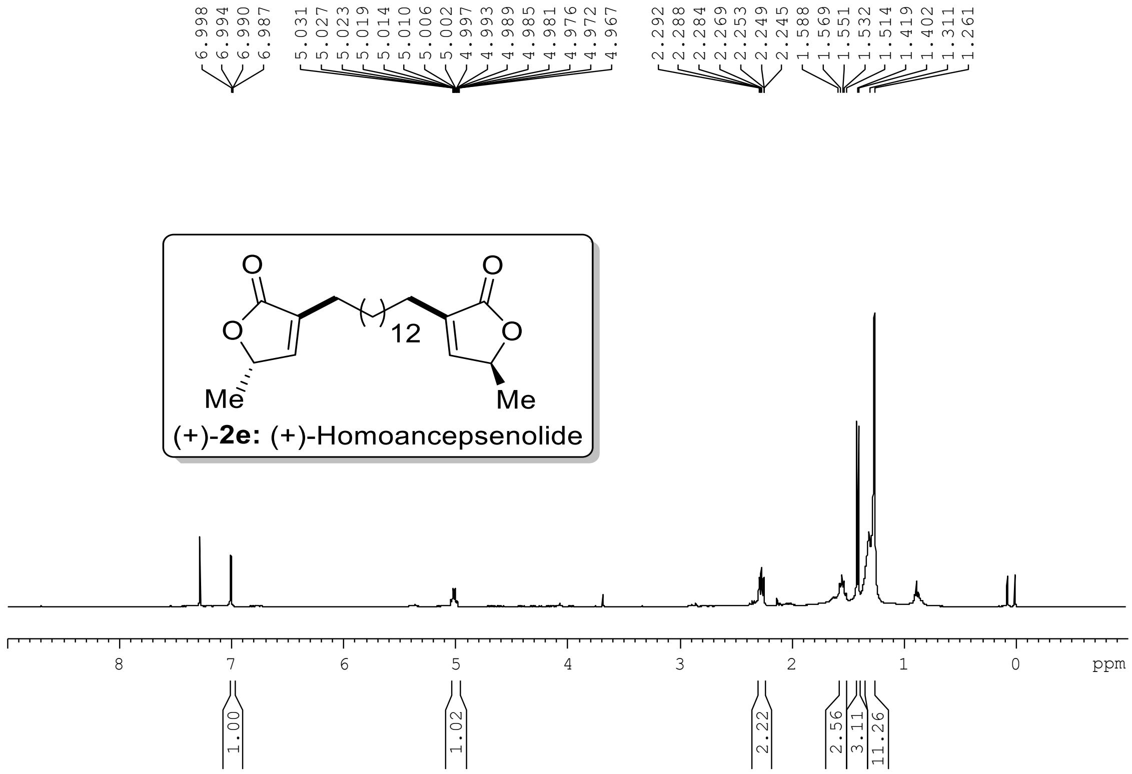

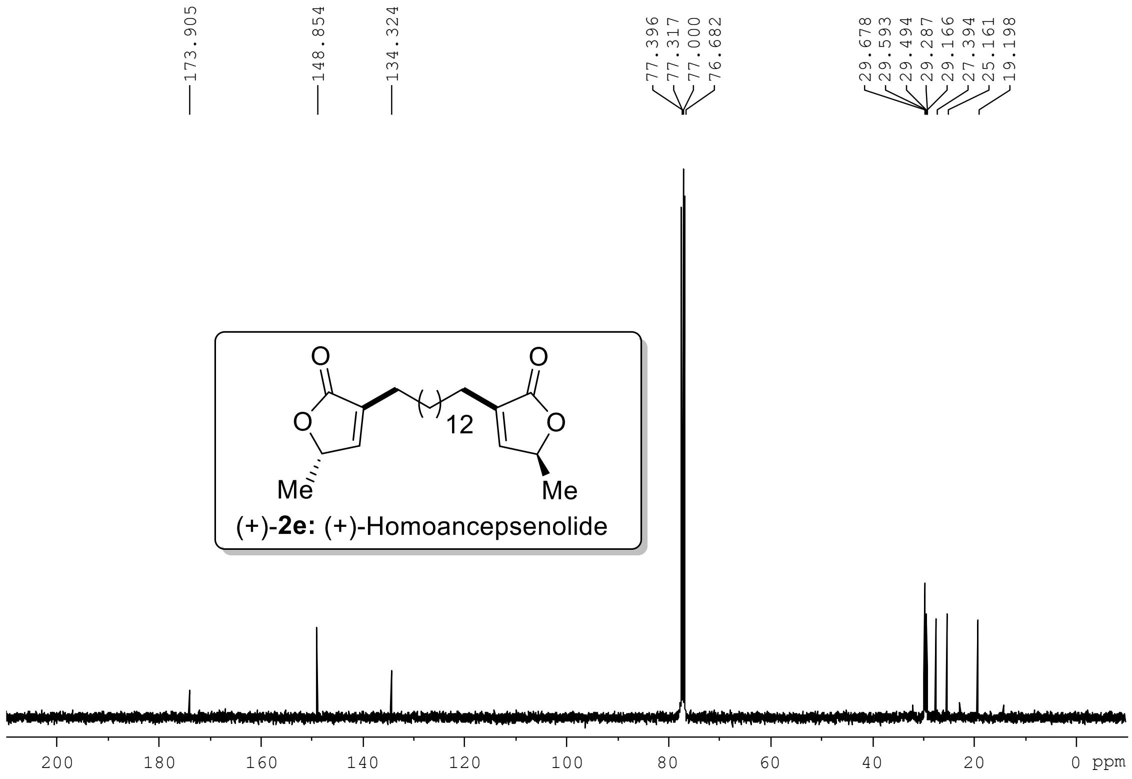

Figure-S5. ^{1}H and ^{13}C NMR spectra of the product (+)-**2e** **[**(+)-Homoancepsenolide].

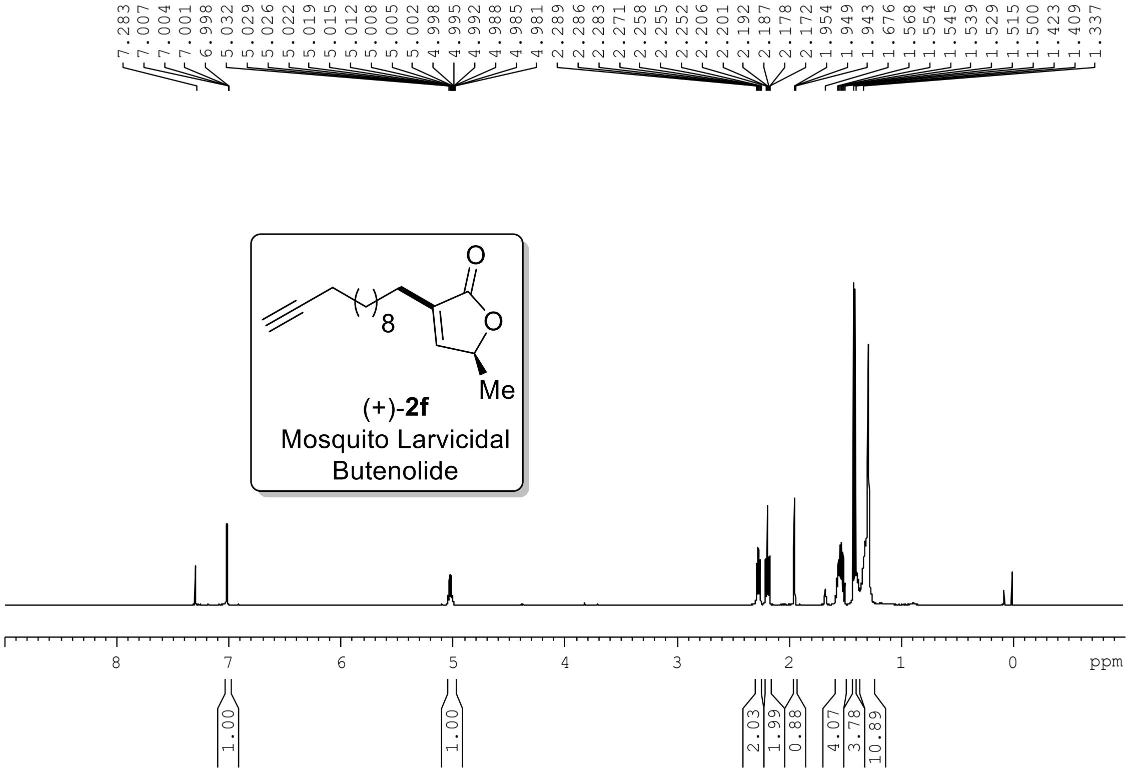

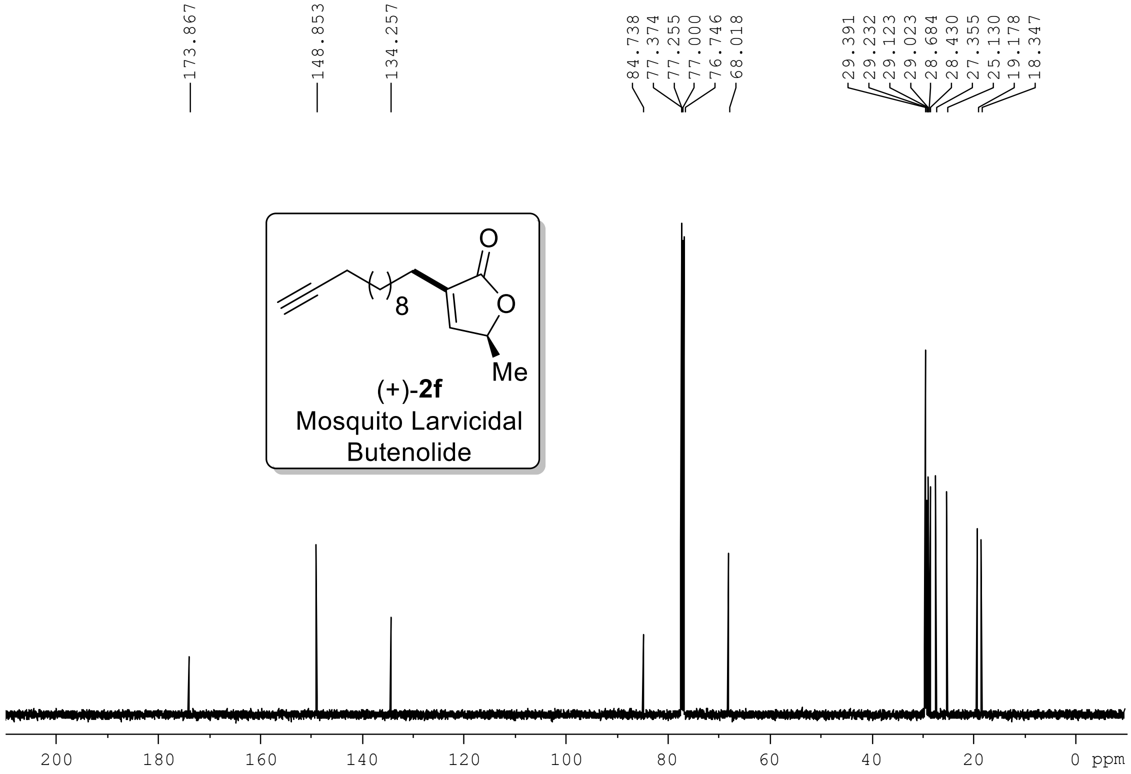

Figure-S6. ^{1}H and ^{13}C NMR spectra of the product **(+)-2f** Mosquito Larvicidal Burenolide.

Isolation and Yield Comparative Tables of Butenolide Natural Products.

Isolation of C-4 Butenolide:[2a]

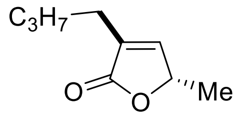

2a

Butenolide, (*S*)-3-butylyl-5-methyl-2[5*H*]furanone is metabolite from *streptomyces griseus*.

Table-S1: Yield comparative table of butenolide (+)-**2a**.

Reference	Starting material	Number of steps	Overall yield	$[\alpha]_D^{25}$
Bernardi *et al*. J. Org. Chem, 1985	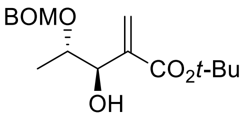	2 steps	unknown	+11.7 (c 0.16, $CHCl_3$)
Nishide *et al*. Tetrahedron 1994	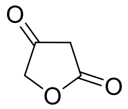	4 steps	53%	+79.2 (c 1.18, $CHCl_3$)
Tsunoda *et al*. Tetrahedron Lett. 2000	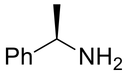	7 steps	11%	Not reported
Yan-Tao He *et al*. Tetrahedron 2002	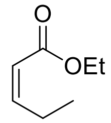	3 steps	69%	+44.3 (c 1.24, $CHCl_3$) with 82% ee
Amonkar *et al*. Synthesis 2005	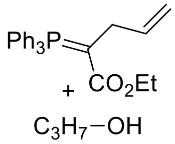	3 steps	40%	Racemic
Ferrarini *et al*. Tetrahedron Lett. 2010	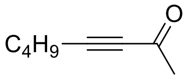	3 steps	51% 94% ee	–
Present OrgRC method	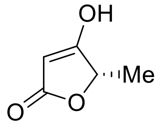	3 steps	43%	+ 36.1 (c 0.28, $CHCl_3$)

References for the synthesis: a) A. Bernardi, M. G. Beretta, L. Colombo, C. Gennari, G. Poli, and C. Scolastico, *J. Org. Chem.* 1985, **50**, 4442; b) K. Nishide, A. Aramata, T. Kamanaka, T. Inoue, and M. Node, *Tetrahedron* 1994, **50**, 8337; c) T. Tsunoda, T. Nishii, M. Yoshizuka, C. Yamasaki, T. Suzuki and S. Ito, *Tetrahedron Lett.* 2000, **41**, 7667; d) Y. –T. He, H. –N. Yang, and Z. –J. Yao, *Tetrahedron* 2002, **58**, 8805; e) C. P. Amonkar, S. G. Tilve, and P. S. Parameswaran, *Synthesis* 2005, 2341; f) R. S. Ferrarini, A. A. Dos Santos, and J. V. Comasseto, *Tetrahedron Lett.* 2010, **51**, 6843.

<u>Isolation of C-6 Butenolide:</u>

Butenolide, (*S*)-3-hexyl-5-methylfuran-2(5*H*)-one is metabolite from *streptomyces griseus*.

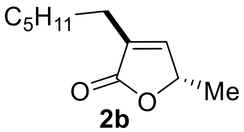

2b

Table-S2: Yield comparative table of butenolide (+)-**2b**.

Reference	Starting material	Number of steps	Overall yield	$[\alpha]_D^{25}$
Amonkar *et al*. Synthesis 2005	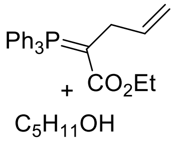	3 steps	49.6%	Racemic
Ferrarini *et al*. Tetrahedron Lett. 2010	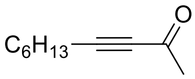	3 steps	51.8% 96% ee	–
Present OrgRC method	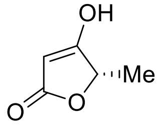	3 steps	48%	+ 24.9 (c 0.28, $CHCl_3$)

<u>References for the synthesis:</u> a) C. P. Amonkar, S. G. Tilve, and P. S. Parameswaran, *Synthesis* 2005, 2341; b) R. S. Ferrarini, A. A. Dos Santos, and J. V. Comasseto, *Tetrahedron Lett.* 2010, **51**, 6843.

<u>Isolation of C-16 Butenolide:</u>[2i]

C-16 butenolide was first isolated from gorgonian *pterogorgia* spp. in 2006 by Lorenzo *et al*. and its enantiomer was isolated from gorgonian *pterogorgia anceps* in 1999 by Guo *et al*. Prior to its isolation C-16 butenolide was reported by Ortuno *et al*. who isolated it as an intermediate while attempting to synthesize 3,4,5-trisubstituted furanones.

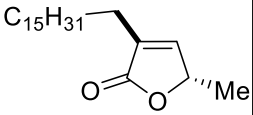

2c

Table-S3: Yield comparative table of butenolide (+)-**2c**.

Reference	Starting material	Number of steps	Overall yield	$[\alpha]_D^{25}$
Ortuno *et al*. Tetrahedron, 1988	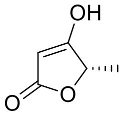	4 steps	25.4%	+26.7 (c 1.3, dioxane)
Present OrgRC method	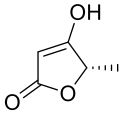	3 steps	54%	+24.85 (c 0.5, $CHCl_3$)

References for the synthesis: R. M. Ortuno, J. Bigorra, and J. Font, *Tetrahedron* 1988, **44**, 5139.

Isolation of Ancepsenolide:[2b]

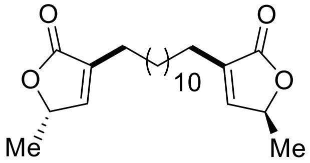

2d (+)-Ancepsenolide

(+)-Ancepsenolide was isolated from gorgonian *pterogorgia anceps*, a marine organism in 1966. Later in 1971, Schimtz *et al*. isolated it from another gorgonion *pterogorgia guadalupensis* and in 1994 Rodriguez *et al*. reported its isolation from *pterogorgia citrina.*

Table-S4: Yield comparative table of butenolide (+)-**2d**.

Reference	Starting material	Number of steps	Overall yield	$[\alpha]_D^{25}$
Podraza, *et al.* J. Nat. Pro., 1985	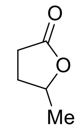	4 steps	19%	Racemic
Larson, *et al*. J. Org. Chem., 1985	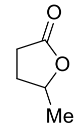	3 steps	77%	Racemic
Trost and Muller J. Am. Chem. Soc., 1994 J. Am. Chem. Soc., 1995	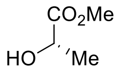	7 steps	31%	+39.6 (c 0.4, $CHCl_3$)
Takai and Iriye Bio. Bio. Bio., 2001	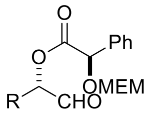	3 steps	10%	+20.0 (c 0.1, $CHCl_3$)
Ghobril *et al*. Eur. J. Org. Chem., 2011	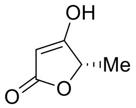	4 steps	12%	+45.53 (c 0.43, $CHCl_3$)
Present OrgRC method	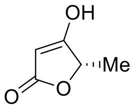	3 steps	55%	+30.5 (c 0.12 , $CHCl_3$)

References for the synthesis: a) K. F. Podraza, and A. T. Sneden, *J. Nat. Prod.* 1985, **48**, 792; b) G. L. Larson, and R. M. Betancourt de Perez, *J. Org. Chem*. 1985, **50**, 5257; c) B. M. Trost, and T. J. J. Muller, *J. Am. Chem. Soc*. 1994, **116**, 4985; d) B. M. Trost, T. J. J. Muller, and J. Martinez, *J. Am. Chem. Soc*. 1995, **117**, 1888; e) K. Takai, and R. Iriye, *Biosci. Biotechnol. Biochem*, 2001, **65**, 1903; f) C. Ghobril, J. Kister, and R. Baati, *Eur. J. Org. Chem.* 2011, 3416.

Isolation of Larvicidal Butenolides:[2h]

Butenolides **2f** was isolated by Ratnayake *et al.* in 2001 from three species of *Hortonia genus* and known to exhibit mosquito larvicidal activity.

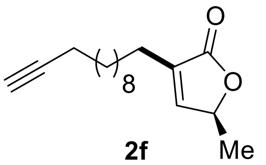

2f
Mosquito Larvicidal Butenolide

Table-S5: Yield comparative table of butenolide (+)-**2f**.

Reference	Starting material	Number of steps	Overall yield	$[\alpha]_D^{25}$
Jiang *et al.* Chinese J. Chem, 2002	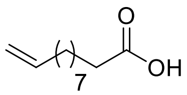	10 steps	7.6%	+30.6 (c 0.5, $CHCl_3$)
Yan-Tao He *et al.* Tetrahedron 2002	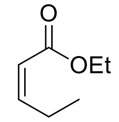	3 steps	46.4%	+30.3 (c 0.4, $CHCl_3$)
Present OrgRC method	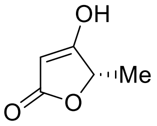	4 steps	30.6%	+30.6 (c = 0.11, $CHCl_3$)

References for the synthesis: a) S. Jiang, Y. –L. Wu, and Z. –J. Yao, *Chinese J. Chem*, 2002, **20**, 692; b) Y. –T. He, H. –N. Yang, and Z. –J. Yao, *Tetrahedron* 2002, **58**, 8805.

References:

1) S. Brandange, L. Flodman, and A. Norberg, *J. Org. Chem.* 1984, **49**, 927-928.
2) For Reviews: (a) Y. S. Rao, *Chem. Rev*., 1964, **64**, 353; (b) D. W. Knight, *Contemp. Org. Synth*., 1994, **1**, 287. For papers see. (a) N. N. Gerber, *Tetrahedron Lett.* 1973, **10**, 771; (b) A. D. Rodrfguez and C. Ramfrez, *J. Nat. Prod.*, 1994, **57**, 339; (c) F.Y.

Dijstra and T. O. Z. Wiken, *Lebensm. Unters. Forsch.* 1973, **160**, 263; (d) M. Mikayado, T. Kato, N. Ohno, H. Yoshioka, and H. Ohshio, *Agric. Biol. Chem.* 1977, **41**, 57; (e) J. Corbera, J. Font, M. Monsalvatje, R. M. Ortuiio and F. Sanchez-Ferrando *J. Org. Chem.* 1988, **53**, 4393; (f) M. D. Esposti, A. Ghelli, M. Ratta, D. Cortes and E. Estornell, *Biochem. J.*, 1994, **301**, 161; (g) Y. –W. Guo, M. Gavagnin, E. Mollo, E. Trivellone and G. Cimino *J. Nat. Prod.* 1999, **62**, 1194; (h) R. Ratnayake, V. Karunaratne, B. M. R. Bandara, V. Kumar, J. K. MacLeod and P. Simmonds, *J. Nat. Prod.*, 2001, **64**, 376; (i) M. Lorenzo, I. Brito, M. Cueto, L. D'Croz and J. Darias, *Org. Lett.* 2006, **8**, 5001.
